# Tumors of the Palate from Hypertrophy of the Salivary Glands

**Published:** 1858-01

**Authors:** Jules Royer, N. H. Byford


### ARTICLE XVIII.

*Tumors of the Palate from Hypertrophy of the Salivary Glands.* By M. JULES ROYER. Translated by N. H. Byford, M. D.

AFTER some remarks on the history of these tumors, and the surgical anatomy of the region in which they occur, the author gives a general description of this variety of glandular hypertrophy. We give only that part of the paper which we regard of most practical importance.

These are more common in young persons of strong constitution and good health ; nearly as frequent among females as males.

Their growth is generally very slow ; they may remain stationary for ten, fifteen, and even twenty years, but sometimes they augment in size very rapidly.

They are almost always located in the *velum palati*, and when found on the vault, they seem to be prolongations of tumors arising in the soft palate. I know of but one case in which the disease was primarily seated in the roof of the mouth. Many patients who have noticed the affection from its origin, designate the upper edge of the soft palate near the median line as the part first affected, and this is precisely the seat of the larger glandular granules. One characteristic of much importance is the complete non-adherence of these tumors to the surrounding parts; they are so completely isolated, in fact, that they may be easily enucleated without trouble or loss of substance to the surrounding tissues. Most frequently the covering mucous membrane completely retains its normal appearance; it is sometimes a little too red, seldom inordinately pale. Sometimes a small vein may be seen issuing from the convexity of the tumor. The mucous membrane is always free, and may be made to glide over the gland, and sometimes even be pinched up into a fold, and it never presents any appearance of ulceration. These tumors form an elevation under the mucous membrane, very definitely marked even to the sight, and which may be traced with great exactitude by the finger. Their consistence is firm and elastic, and on the surface they are mamelonated or lobulated. They project very little above the surface when situated in the velum, owing to the small resistance of their muscular or aponeurotic cushion, but upon the root of the tongue they often obtain sufficient prominence to raise up the soft palate. Tumors situated near the median line do not pass through the fibrous partition depending from the spinous press of the ossa palati, but so far pushes it and the uvula to one side as to *appear* to occupy both sides of the palate. They are not painful even with considerable pressure, and the reason for applying for surgical aid is that the patients experience difficulty from the rise of the tumor in swallowing, breathing, or speaking. As their growth is very slow, so may they acquire considerable volume without attracting attention—deglutition, phonation, and respiration being unaffected. The symptom which most generally declares itself first, is an alteration in the tone of the voice, but by the patient it is nearly always attributed to some other cause than the right one. A distinction between these and cancerous tumors of the palate is most easy. These last are developed more rapidly, the mucous membrane is traversed by numerous varicose veins, they are attended by lancinating pain. The last symptom is seldom absent when they have been in existence for any considerable length of time. They produce engorgement and degeneration in the neighboring lymphatic gangliars, and produce the general appearance known as the cancerous cachexia. Encephaloid cancer is soft, and often apparently fluctuates to the touch. Scirrhus, although hard and firm like the benign glandular tumor, adheres to the mucous membrane, and induces ulceration of the surface.

Syphilitic tumors, which occur in the tertiary form of this affection, might be mistaken for these hypertrophic nodules, but their progress is different. They are not so slow of formation and development; but an especial difference is, that their boundaries are not so definite as the glandular tumors. Other portions of the body are also affected similarly, and often there is syphilitic cachexia. The history, too, is essentially that of syphilis.

These glandular tumors are not dangerous to life, but if let alone, by the considerable volume they attain, cause functional derangement calling for their removal. Their ablation is never followed by a return of the disease.

It is hardly necessary to say that their treatment is essentially surgical, and consists of their excision.

The operation, which is intended merely to disembarrass the patient, is quite simple—a simple longitudinal incision over the middle of the tumor, separation of the flaps and enucleation are all usually needed. Their feeble adhesion permits this last to be done easily by the finger or spatula. A crucial T or circular incision in rare instances may be required. In one case the excision was not complete, and M. Nelaton applied the actual cautery to the remaining portion.

I saw the patient a year afterward, and the tumor had entirely disappeared. In case from any cause the excision should not be complete, this treatment should be resorted to for fear of recurrence. The loss of blood is generally trifling. If sufficient to require attention, however, the cold lotions, ice, and other surgical remedies for hemorrhage will easily succeed in arresting it. The surgical wound required for extirpation heals kindly, and requires no extraordinary treatment.

A section of one of these tumors presents to the naked eye a yellowish gray, or grayish colored surface. The section at first sight seems smooth and fibrous, but upon close examination, and especially if a lens is used, it shows a furrowed appearance which divides it into lobes, and still smaller rubi divide it into lobules. Under pressure a limpid thin fluid is expelled, as also a more consistent but soft and cheesy-looking substance may be obtained.

This liquid and substance both contain epithelial cells visible by the microscope. The tumor examined as a whole is enveloped by a dense resisting membrane in which it is encisted, and which appears to be formed of lamilated areolar tissue. Very few vessels can be observed either on the surface or in the interior, even with the aid of a microscope. The microscopic character of these tumors has been made out almost entirely from the observation of M. Charles Robin, and consist of the following items of composition, viz.

1st. Hypertrophied glandular *culs de sac*, very numerous and obvious.

2d. Nuclea of epithelial cells, single and aggregated.

3d. Epithelial cells containing nuclea.

4th. Fribro-plastic elements, and fibrous tracti.

5th. In some tumors small calcula are often met with, formed of one or more of the salts of lime.

After this description M. Royer gives more extended information on the histology of these tumors, and closes his work with six cases taken from the hospital practice of Professors Velpeau and Nelaton, and a summary of the main facts relative to six other cases recorded in other scientific collections.—*Gaz. des. Hos.—In Nashville Journal of Medicine and Surgery.*

